# Potentiation of immunomodulatory antibodies with oncolytic viruses for therapy of poorly-immunogenic tumors

**DOI:** 10.1186/2051-1426-2-S3-P132

**Published:** 2014-11-06

**Authors:** Dmitriy Zamarin, Rikke B Holmgaard, Tamar Plitt, James Allison, Taha Merghoub, Jedd D Wolchok

**Affiliations:** 1 Memorial Sloan Kettering Cancer Center, Rego Park, NY, USA; 2 MD Anderson Cancer Center, Houston, TX, USA

## 

Cancer immunotherapy with antibodies targeting immune checkpoints has demonstrated durable clinical benefit, though responses have not been universal and, in particular, not effective for tumors lacking preexisting lymphocytic infiltration. Oncolytic viruses (OV) have recently emerged as an important addition to the immunotherapy armamentarium, with promising activity seen in recent clinical trials. To test whether the inflammatory responses activated by OV could be harnessed to drive therapeutic efficacy of immunomodulatory antibodies in poorly-inflamed tumors, we employed poorly immunogenic animal tumor models, including the transplantable B16-F10 and TRAMP C2 tumors and genetically-engineered BRAF^V600E^PTEN^fl/fl ^melanoma. As a model OV, we used Newcastle Disease Virus (NDV), an avian virus that was previously demonstrated to be a strong activator of type I interferon and DC maturation. Administration of NDV to a single tumor in animals bearing bilateral flank tumors concomitant with adoptive transfer of naïve transgenic antigen-specific CD4 or CD8 lymphocytes led to efficient infiltration of bilateral tumors with the adoptively-transferred cells, an effect that was not seen in the absence of virus treatment or when heterologous tumors were used on both flanks. Consistent with these findings, therapy with NDV in the absence of adoptive T cell transfer resulted in lymphocyte infiltration and tumor growth delay in both virus-injected and distant tumors in all tumor models. Therapy with NDV was associated with upregulation of PD-L1 on tumor cells and tumor-infiltrating innate and adaptive immune cells in both virus-injected and distant tumors, an effect that was in part mediated by type I IFN produced during viral infection. Notably, combination therapy with localized NDV and systemic blockade of PD-1, PD-L1, or CTLA-4 led to rejection of the majority of both virus-injected and distant tumors in the B16-F10 and TRAMP C2 models and significant tumor growth delay in the BRAF^V600E^PTEN^fl/fl ^model. This effect was associated with marked tumor infiltration with activated effector, but not regulatory T cells, and was abrogated with CD8 or IFN-γ depletion or in IFNAR^-/- ^mice. These results demonstrate that in poorly-immunogenic cancers lacking TIL infiltration, therapy with oncolytic viruses such as NDV can convert the tumors to an inflamed phenotype in absence of virus spread to all tumor sites, resulting in potentiation of therapeutic efficacy of antibodies targeting immune checkpoints. These findings provide a rationale for further exploration of such combination strategies, and trials using different OVs are currently in development to test this concept in clinic.

